# Phenotypic effects of the Y chromosome are variable and structured in hybrids among house mouse recombinant lines

**DOI:** 10.1002/ece3.5196

**Published:** 2019-05-07

**Authors:** Iva Martincová, Ľudovít Ďureje, Jakub Kreisinger, Miloš Macholán, Jaroslav Piálek

**Affiliations:** ^1^ Research Facility Studenec, Institute of Vertebrate Biology Czech Academy of Sciences Brno Czech Republic; ^2^ Department of Botany and Zoology, Faculty of Science Masaryk University Brno Czech Republic; ^3^ Department of Zoology, Faculty of Science Charles University in Prague Prague Czech Republic; ^4^ Laboratory of Mammalian Evolutionary Genetics, Institute of Animal Physiology and Genetics Czech Academy of Sciences Brno Czech Republic

**Keywords:** *Mus musculus domesticus*, *Mus musculus musculus*, phenotype variation, sperm quality, wild‐derived strain, Y‐associated effects

## Abstract

Hybrid zones between divergent populations sieve genomes into blocks that introgress across the zone, and blocks that do not, depending on selection between interacting genes. Consistent with Haldane's rule, the Y chromosome has been considered counterselected and hence not to introgress across the European house mouse hybrid zone. However, recent studies detected massive invasion of *M. m. musculus* Y chromosomes into *M. m. domesticus* territory. To understand mechanisms facilitating Y spread, we created 31 recombinant lines from eight wild‐derived strains representing four localities within the two mouse subspecies. These lines were reciprocally crossed and resulting F1 hybrid males scored for five phenotypic traits associated with male fitness. Molecular analyses of 51 Y‐linked SNPs attributed ~50% of genetic variation to differences between the subspecies and 8% to differentiation within both taxa. A striking proportion, 21% (frequencies of sperm head abnormalities) and 42% (frequencies of sperm tail dissociations), of phenotypic variation was explained by geographic Y chromosome variants. Our crossing design allowed this explanatory power to be examined across a hierarchical scale from subspecific to local intrastrain effects. We found that divergence and variation were expressed diversely in different phenotypic traits and varied across the whole hierarchical scale. This finding adds another dimension of complexity to studies of Y introgression not only across the house mouse hybrid zone but potentially also in other contact zones.

## 1 INTRODUCTION

Secondary contact hybrid zones where genetically diverged taxa meet and mate are widely present across the Tree of Life. Following initial contact, genes that have diverged in allopatry are brought into novel combinations and tested for their effects on fitness. Natural selection will sieve the interacting genomes, incompatible combinations will be selected against and confined to the hybrid zone, neutral genes will diffuse freely to both sites, and selectively advantageous variants will recombine away from their native genomes and spread across the border into non‐native genetic background (Barton & Gale, 1990; Payseur, 2010). Faster evolution of X chromosomes (Coyne & Orr, 1989; Presgraves, 2008), meiotic recombination restricted to an X‐Y pairing region of sex chromosomes (Burgoyne, 1982), and their lower effective sizes relative to autosomes (Hedrick, 1985) imply gene flow of sex‐linked loci across the zone to be severely restricted. In addition, according to Haldane's rule (Haldane, 1922), sterility or inviability of hybrids should occur, primarily, in males and hence Y chromosome introgression should be particularly affected.

However, empiric studies on the behavior of Y chromosomes in contact zones have yielded inconsistent results. For example, none or extremely limited introgression of Y chromosomes was found in hybrid zones between two subspecies of the European rabbit, *Oryctolagus cuniculus* (Carneiro et al., 2013; Geraldes, Carniero, et al., 2008)*,* shrew species *Sorex araneus* and *S. antinorii* (Yannic, Basset, & Hausser, 2008), two evolutionary lineages of the common vole, *Microtus arvalis* (Beysard & Heckel, 2014), and between two lineages of the field vole, *Microtus agrestis* (Beysard, Perrin, Jaarola, Heckel, & Vogel, 2012). On the other hand, whereas the vast majority of autosomal and mitochondrial genes were found to change rapidly in a hybrid zone between the baboon species *Papio kindae* and *Papio griseipes*, the Y chromosome of *P. kindae* showed extensive introgression into *P. griseipes* territory (Chiou, 2017). Introgressive hybridization was reported also between two North American deer species (*Odocoileus virginianus* and *O. hemionus*) where *virgianus* alleles of the Y‐linked *Zfy* gene spread into *hemionus* genome (Wheeldon, Rutledge, Patterson, White, & Wilson, 2013).

Perhaps the most intriguing case of the Y introgression has been reported from the European house mouse hybrid zone between two subspecies, *Mus musculus musculus* and *M. m. domesticus*. After their split 0.5–1 million years ago (Duvaux, Belkhir, Boulesteix, & Boursot, 2011; Geraldes, Basset, et al., 2008), the two taxa have colonized Europe following different routes. The *musculus* mice migrated across the plains north of the Black Sea and now inhabit the northern and eastern part of Europe. The *domesticus* mice moved from the Middle East through Asia Minor and eastern Mediterranean to the southern and western part of the continent (Cucchi, Auffray, & Vigne, 2012). Where they meet the two subspecies form a more than 2,500‐km‐long and only ~20‐km‐wide secondary hybrid zone stretching from Norway to Bulgaria (Ďureje, Macholán, Baird, & Piálek, 2012; Jones, Kooij, Solheim, & Searle, 2010). The zone has been studied across several distant geographic transects, and there is compelling evidence that it represents a semipermeable barrier allowing neutral diffusion of some genes (e.g., mtDNA) while hampering introgression of other parts of the genome (e.g., the central X chromosome) (Payseur, Krenz, & Nachman, 2004, Macholán et al., 2007, 2011, Dufková, Macholán, & Piálek, 2011, Janoušek et al., 2012).

Whereas early studies from distant parts of the hybrid zone such as Bulgaria (Vanlerberghe, Dod, Boursot, Bellis, & Bonhomme, 1986), southern Bavaria (Tucker, Sage, Warner, Wilson, & Eicher, 1992), and Denmark (Dod et al., 1993) showed strongly impeded movement of Y chromosomes across the zone, massive introgression of *musculus* Y into *domesticus* autosomal background was reported in the Czech/German transect (Macholán et al., 2008; Munclinger, Brozikova, Sugerkova, Pialek, & Macholan, 2002). A subsequent study from the same region revealed sperm count restoration in hybrids with introgressed *musculus* Y chromosomes (Albrechtova et al., 2012). Moreover, Y introgression is not restricted to this area. Analysis of 224 localities in Central Europe revealed that the *musculus* Y invades across the zone in multiple replicates and that this introgression is essentially unidirectional (Ďureje et al., 2012). The presence of *musculus* Y chromosomes in the *domesticus* territory was also reported from Scandinavia (Jones et al., 2010). However, except the study of sperm‐related traits by Albrechtová et al. (2012), data inferring the dynamics of the *musculus* Y chromosome spread in a wider geographic context and its possible phenotypic correlates are lacking.

The key question to be answered is then whether there are any intersubspecific differences in phenotypes associated with Y chromosome that affect male fitness. However, reducing the search for differences in phenotypic traits to the intersubspecific level can lead to biased inference on Y spread dynamics. For example, if different localities within the subspecies display significant variation in the Y‐associated phenotypes, their effects can be canceled out when averaged, and hence, this variability will be obscured. Yet the intrasubspecific variation creates local minima and maxima in the fitness landscape and thus can drive the spread of beneficial Y‐linked variants across the subspecies range(s) as well as across the hybrid zone. In general, if Y chromosome variation is geographically structured and associated with variation in phenotypic traits, we might expect three alternative outcomes with respect to the Y introgression ability in distant replicates of the house mouse hybrid zone. First, there will be systematic asymmetric intersubspecific Y‐linked effects causing directional differences in fitness and this result will suggest uniformity of the Y introgression in the direction of the advantageous Y variant. Second, detection of intrasubspecific polymorphism in Y effects at various geographic regions will be indicative for different behavior of the Y within or across various replicates of the zone. Third, low phenotypic variance explained by geographically structured Y chromosome variation for a particular phenotypic trait will indicate the absence of association between the Y and a phenotype. In this case, the behavior of the Y in the hybrid zone and/or within the subspecies will be subject to neutral or random processes.

To test the three alternatives conditioning the Y spread, we conducted a study in which sampling was designed to evaluate effects of the Y chromosome both at intra‐ and intersubspecific levels. Data were obtained from reciprocal F1 hybrids derived experimentally in the laboratory. Polymorphism was introduced by crossing eight wild‐derived mouse strains and mixing their genomes in a hierarchic way that allowed us to infer Y effects from the within‐strain to intersubspecific level. Fitness has many components and each of them can be affected by introduced polymorphism in different ways. As we documented that sperm‐related traits can affect the dynamics of the Y spread (Albrechtová et al. 2012), we focused on sperm quantity and quality (including number of sperm heads dissociated from tail and number of abnormal sperm heads). Using experimental animals allowed us to control for age and hence explicitly remove allometric relationships between body traits contributing to male–male competition. Consequently, we added also body and testis size to analysis.

## 2 MATERIAL AND METHODS

### 2.1 Animals and experimental design

The mouse strains under study have been developed and maintained in the breeding facility of the Institute of Vertebrate Biology in Studenec (licenses for keeping and experimental work 61,974/2017‐MZE‐17214 and 62,065/2017‐MZE‐17214, respectively). Four strains represented *domesticus*: The STRA and STRB with more than 20 generations of brother–sister mating were inbred (Piálek et al., 2008), whereas SCHUNT and SCHEFE were in the 13th and 14th generation of inbreeding, respectively. The four strains representing *musculus*, BUSNA, BULS, STUS, and STUF, were fully inbred (Piálek et al., 2008). To capture local variation, two strains per locality have been derived. Specifically, STRA and STRB originate from Straas [N: 50°11′, E: 11°46′], SCHUNT and SCHEFE from Schweben [N: 50°26′, E: 9°35′], both from Germany, BULS and BUSNA from Buškovice [N: 50°13′, E: 13°23′ and N: 50°14′, E: 13°22′, respectively], and STUS and STUF from Studenec [N: 49°12′, E: 16°04′], both from the Czech Republic.

**Figure 1:**
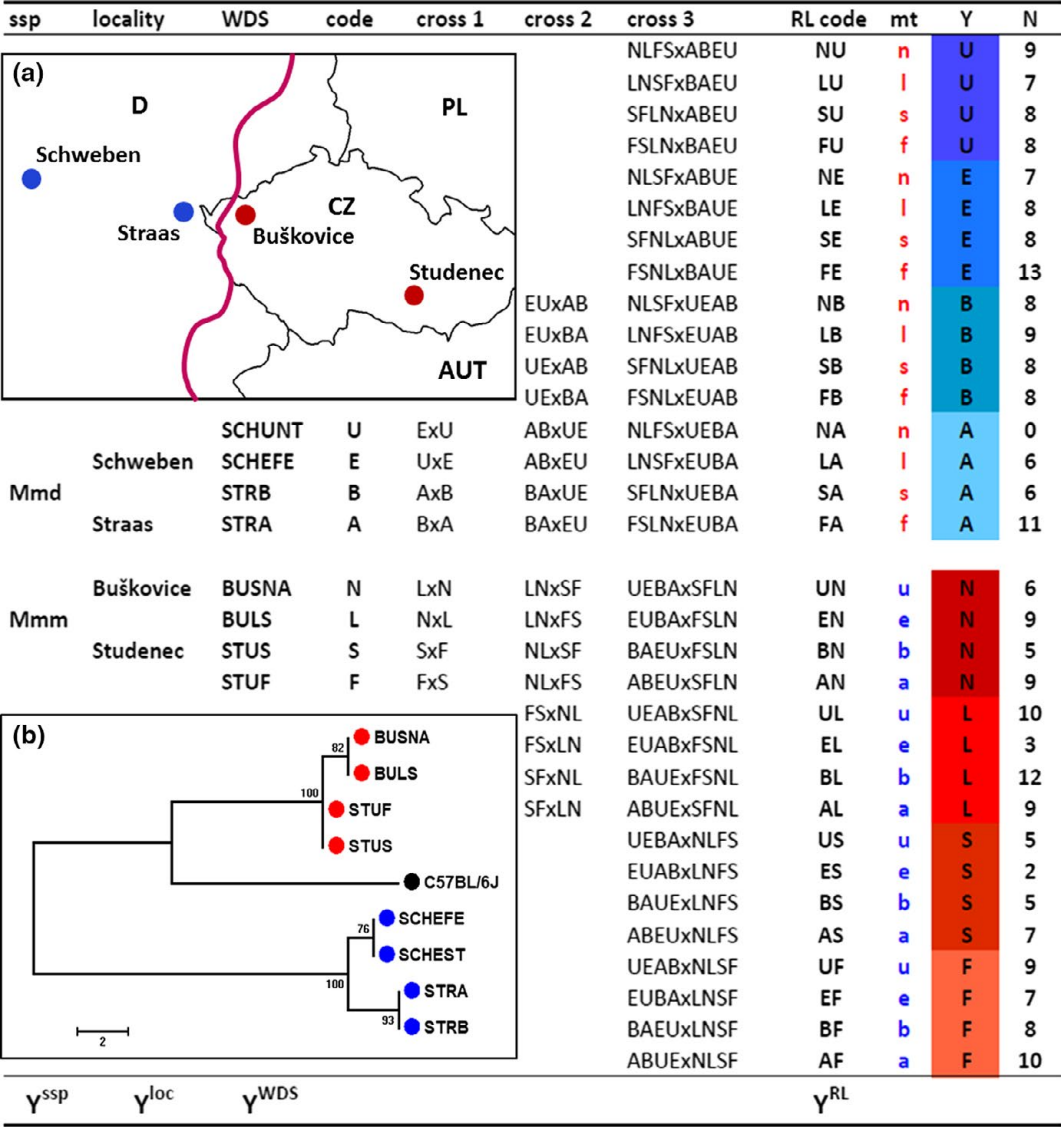
The design of the experimental crossing. The first column lists subspecies, the second localities and the third, full names of founder wild‐derived strains used for the crossing. Each wild‐derived strain is also labeled with a one‐letter code (fourth column) for simplicity. In all crosses, the first letter stands for a female and the second letter for a male. The RL codes consist of two letters, the first one indicating the origin of mtDNA (column mt) and the second one pointing to the Y chromosome origin in each cross (column Y). The Y column displays also color codes used in bar plots, with blue hues for *domesticus* and red hues for *musculus* mice. Autosomal and X‐linked genomes are mixtures of *domesticus* and *musculus* genomes. *N* gives the numbers of tested males per cross. The bottom line indicates levels at which Y chromosomes were tested for phenotypic effects. Inserted panel A depicts a map of trapping localities of the founders of 8 wild‐derived strains studied. The violet line indicates the house mouse hybrid zone course. Panel B shows the neighbor‐Joining tree of Y chromosome haplotypes based on 51 SNPs (see the text). The C57BL/6J strain represents a reference Y haplotype. The bootstrap values based on 100 replicates are shown at each node. The scale is in numbers of SNPs distinguishing pairs of strains

The sampling of localities, from which in total more than 25 strains were developed, was designed a priori to mirror the increase of genetic variation with growing distance from the zone. The selection of eight strains was conditioned by statistical models used to test Y effects on phenotypes at various geographically structured levels (detailed below). The sampled localities are located symmetrically about 50 and 250 km from the estimated hybrid zone center (Figure 1a).

The parental strains were crossed in a combinatory design as depicted in Figure 1. The design reflects polymorphism introduced by mating direction, geographic origin (strain and locality effects), and subspecific status of each strain. The mating scheme started with crossing strains within each locality (Figure 1, cross 1), then between localities within the subspecies (Figure 1, cross 2), and finally, between the subspecies (Figure 1, cross 3). As a result, 32 recombinant lines (RL) are altogether expected from the experimental mating scheme.

### 2.2 Phenotyping

**Figure 2:**
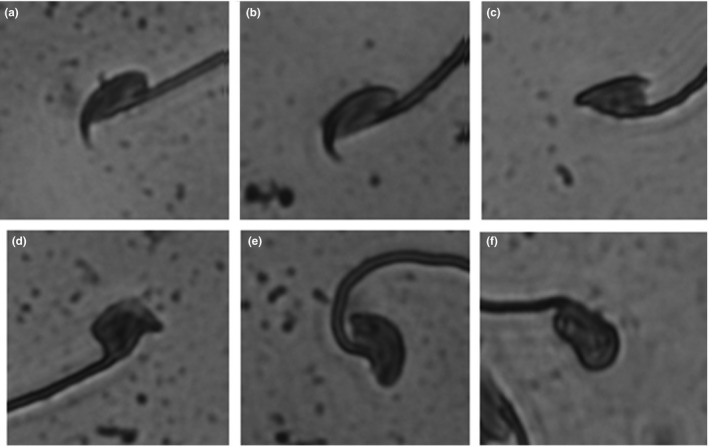
The classification of normal and abnormal sperm heads. The first two figures represent normal sperm heads of a *domesticus* (a) and a *musculus* (b) male. Figures c–f show examples of abnormal sperm heads

We succeeded in generating experimental males from 31 RLs. In total, 240 males were examined. Males were sacrificed by cervical dislocation and dissected at 60 days of age. One external measurement, body weight (BW, to 0.01 g), was taken. The spleen was removed, weighed, and preserved in 96% ethanol for molecular analysis. The testes were weighed individually using analytical balances (TW, to 0.0001 g), and the values were averaged. Spermatozoa were released from the whole left epididymis, and the number of sperm heads was counted in ten squares of a Bürker chamber using an Olympus CX41 microscope under 200× magnification (for details see Vyskočilová, Trachtulec, Forejt, & Piálek, 2005). The mean value was then used as a representative of the individual's sperm count (SC). The frequency of dissociated sperm heads (DSH) was estimated from five squares. Variation in the sperm head shape (ASH) was treated as a binomial variable with heads classified either as normal (Figure 2a,b) or as abnormal (Figure 2c–f). The proportion of ASH used for statistical analyses was estimated from 3 squares.

### 2.3 Molecular analysis

Genetic divergence of Y chromosomes in the eight strains (Figure 1b) was inferred from a set of 51 SNPs spread along the short arm between the *Zfy1* and *Sry* genes. This set is a part of the high‐density Mouse Diversity Array (MDA) containing 623,124 SNPs designed to capture genetic variation present in the laboratory mice (Yang et al., 2009). Genotypes for six strains (STRA, STRB, BUSNA, BULS, STUS, and STUF) and the C57BL/6J strain were obtained from Yang et al. (2011). The SCHEST and SCHWEBEN males were genotyped separately using the same MDA probe sets (Yang et al., 2009). The evolutionary history of the Y chromosome and the robustness of the resulting tree was inferred using the neighbor‐Joining method (Saitou & Nei, 1987) and bootstrap resampling (Felsenstein, 1985) with 100 replicates as implemented in the MEGA program (Kumar, Stecher, & Tamura, 2016).

### 2.4 Statistical analyses

Statistical analyses were performed in the R statistical environment (RStudio Team, 2015). We used two different approaches. The first one focused on partitioning phenotypic variance associated with the Y effect of subspecies, localities, strains, and RLs. The variance was estimated via hierarchical mixed effect models with Gaussian error structure and fitted with the *lmer* function from the R package lme4 (Bates et al., 2014). Localities, Strains, and RLs were included as nested random effects. Since the subspecies identity of the mice under study had only two states (*musculus* and *domesticus*), resulting variance estimates of corresponding random effects may be imprecise (Crawley, 2002). Therefore, we considered the subspecies category as a fixed predictor. We calculated an *R* ^2^‐like statistics for each model term using the approach described in Nakagawa and Schielzeth (2013). In addition, we estimated parameters for fixed effects and standard deviations for each random effect level. The parametric bootstrap was used to derive corresponding 95% confidence intervals. The random effects were visualized using sjPlot package (Lüdecke, 2018). The distribution of residuals and the presence of outliers were checked using standard diagnostic plots. Where necessary, variables were Box‐Cox transformed (Box & Cox, 1982).

In the second approach, experimental males’ data were partitioned in a downward sequence and compared stepwise based on the origin of their Y chromosomes as specified in Figure 1. We started from the top by testing phenotypic effects that can be attributed to Ys grouped according to their subspecific origin (Y^ssp^ level). Then, we split the data with respect to their Y^ssp^ origin and estimated differentiation between localities within each subspecies (Y^loc^ level). Subsequently, we subdivided the data according to the Y^loc^ level and compared strains within each locality (Y^WDS^ level). Finally, we assessed variation among the recombinant lines sharing Y chromosomes of the same strain origin (Y^RL^; see the Y column in Figure 1). In summary, we performed 15 tests: one test at the Y^ssp^ level, two tests at the Y^loc^ level, four tests at the Y^WDS^ level, and eight tests at the Y^RL^ level. Statistical analyses of inter‐ and intrasubspecific variation followed standard recommendations (Sokal & Rohlf, 1995). TW, BW and SC were distributed normally (Shapiro–Wilk test, *p* > 0.05) and had homogeneous variances (Bartlett test, *p* > 0.05). To compare groups of these variables, we used parametric tests: Welch's two samples *t* test and, at the Y^RL^ level, one‐way ANOVA supplemented with Tukey's *post hoc* test. Two frequency variables, DSH and ASH, were not distributed normally, and hence, they were analyzed by the non‐parametric Wilcoxon and Kruskal–Wallis tests, supplemented with the Nemenyi *post hoc* test at the Y^RL^ level. Type I error was set to 0.05; however, as we performed 15 subsidiary comparisons among means across all hierarchical levels, the significances were Bonferroni corrected (i.e., α = 0.05/15 = 0.003). For the sake of transparency, we report both the uncorrected and corrected significances throughout the forthcoming text.

## 3 RESULTS

### 3.1 Molecular analysis is consistent with geography

Genotypes at 51 SNPs are shown in Data S1. The phylogenetic tree suggests that variation at the Y‐linked loci reflects the geographic position of localities at which the founder mice were collected (cf. Figure 1a and b). Specifically, out of 51 loci scored, 24 SNPs (47%) were fixed for subspecies‐specific variants. The Straas males shared two private SNPs, Buškovice and Schweben males possessed one private SNP, whereas males from Studenec had none. No SNPs were detected between pairs of strains from the same localities.

### 3.2 Explained phenotypic variation at different hierarchical levels.

Phenotype data are available in Data S2. The R script for all statistical analyses including visualization of results is available in Data S3. Partitioning of the overall phenotypic variation attributed to the Y chromosome and corresponding *R* squared‐like values rendered by the hierarchical model (see Methods) is summarized in Table 1. The largest proportion of variance in all variables is represented by the residual effects and hence remains unexplained. The explained variances range between 20.6% and 42.3% for abnormal and dissociated head sperm, respectively, and are disproportionally partitioned across the hierarchical levels. The largest fraction of the explained variability is present at the Y^RL^ level, except for the DSH frequency, where higher variance is explained at the Y^ssp^ level. In SC and TW, the amount of the explained variance at the Y^loc^ level nearly reaches the Y^RL^ level whereas it is zero or negligible at the Y^WDS^ and Y^ssp^ level, respectively. For two remaining variables, BW and ASH, the proportion of explained variance increases with increasing refinement of the tested model, that is, from the Y^ssp^ to Y^RL^ level, with exception of the Y^loc^, where no variance is explained. Details of GLMM estimates (parameter estimates for fixed effects and variances for random effects), their 95% bootstrap confidence, and R squared‐like statistics for each model term are listed in Data S4.

**Table 1:** Estimates of phenotypic variance explained at individual hierarchical levels

	BW		SC		TW		DSH		ASH	
	Variance	*R* ^2^	Variance	*R* ^2^	Variance	*R* ^2^	Variance	*R* ^2^	Variance	*R* ^2^
Y^ssp^	0.011	0.002	0.259	0.008	1.212	0.005	**0.057**	**0.334**	0.022	0.039
Y^loc^	0.000	0.000	5.531	0.161	39.475	0.163	0.000	0.000	0.000	0.000
Y^WDS^	0.336	0.053	0.000	0.000	0.000	0.000	0.000	0.000	0.029	0.052
Y^RL^	**1.098**	**0.173**	**6.375**	**0.186**	**51.716**	**0.214**	0.015	0.089	**0.064**	**0.114**
Residual	4.891	0.772	22.100	0.645	149.752	0.618	0.098	0.577	0.446	0.794

Note

Individual *R* ^2^ is calculated as proportions of overall variance in the model and their sum is 1. Figures in bold indicate maxima of variance explained across the four different hierarchical levels.

### 3.3 Intersubspecific effects of Y (Y^ssp^ level)

All descriptive statistics (means, medians, standard deviations or Q1‐Q3 interquartile values, and significances without and with the Bonferroni correction) across all hierarchical levels are shown in Table S5.

**Figure 3:**
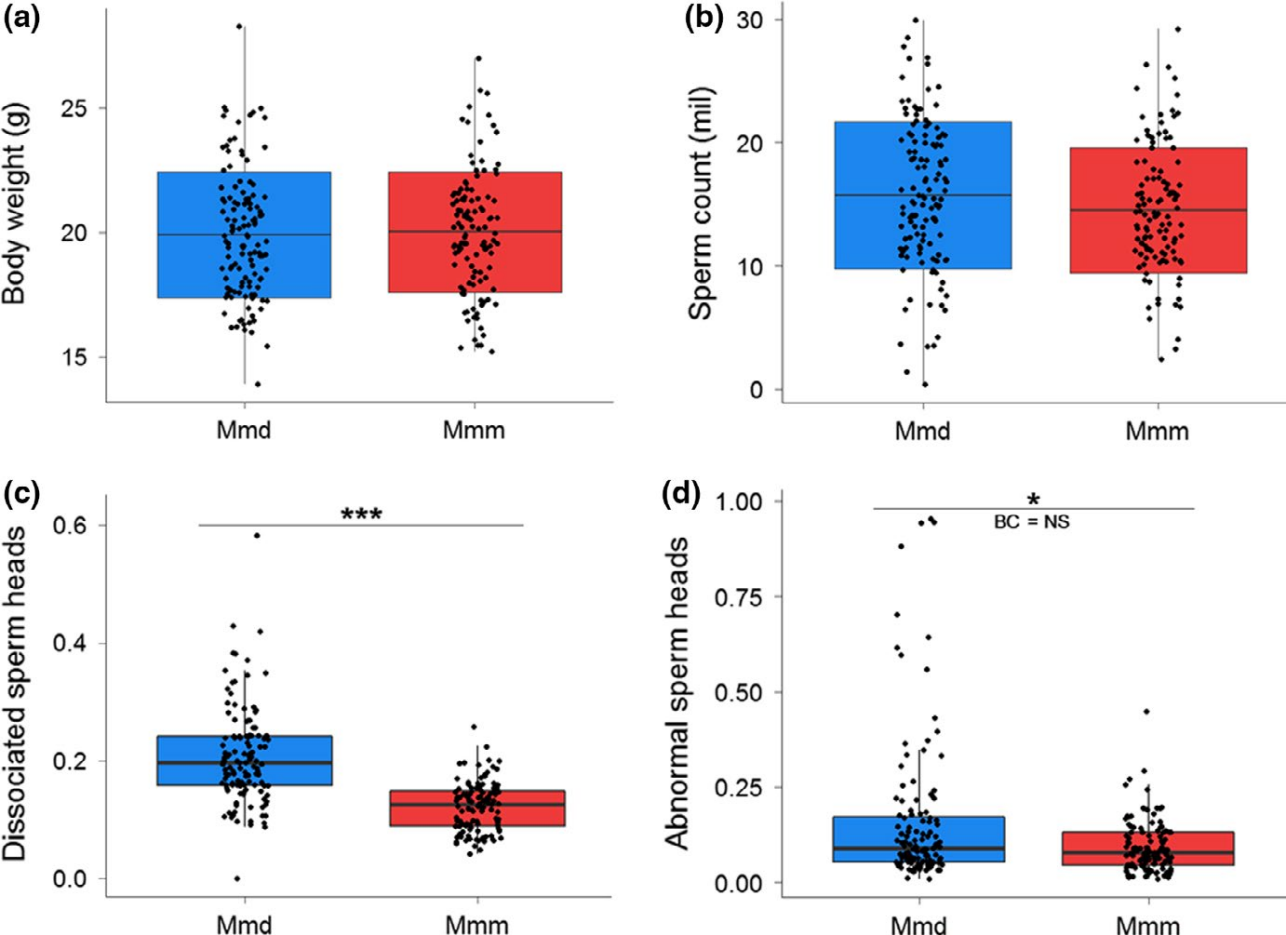
Boxplots for (a) body weight, (b) sperm count, (c) frequency of dissociated sperm heads, and (d) frequency of abnormally shaped sperm heads among male groups with subspecies‐specific types of the Y. In graphs a and b, the crossbars indicate the mean values, the box ranges display standard deviation, and whiskers give ranges between the maximum and minimum values. In graphs c and d, the crossbars refer to the medians, the boxes display the range between the 1st and 3rd quartile, and whiskers span the 1.5 ranges between the lower and upper quartiles. Black dots represent individual values. Lines and asterisks above the boxplots mark uncorrected significant differences between groups of males: “***” 0.001 “**” 0.01 “*” 0.5, BC = NS is displayed when such difference is not significant after the Bonferroni correction

Pronounced intersubspecific genetic divergence in Y chromosomes was only partially associated with differentiation of phenotypic traits. No effect of the Y on body or testis weight and the sperm count was detected (BW and SC are shown in Figure 3a–b, data for TW are in Data S5). However, the proportion of dissociated sperm heads (DSH) showed a different picture: The Y*^domesticus^* males had a significantly higher frequency of DSH than the Y*^musculus^* males. A similar difference was revealed in ASH although its significance (*p* = 0.014) disappeared after the Bonferroni correction (Figure 3c–d).

### 3.4 Intrasubspecific effects (Y^loc^ level)

**Figure 4:**
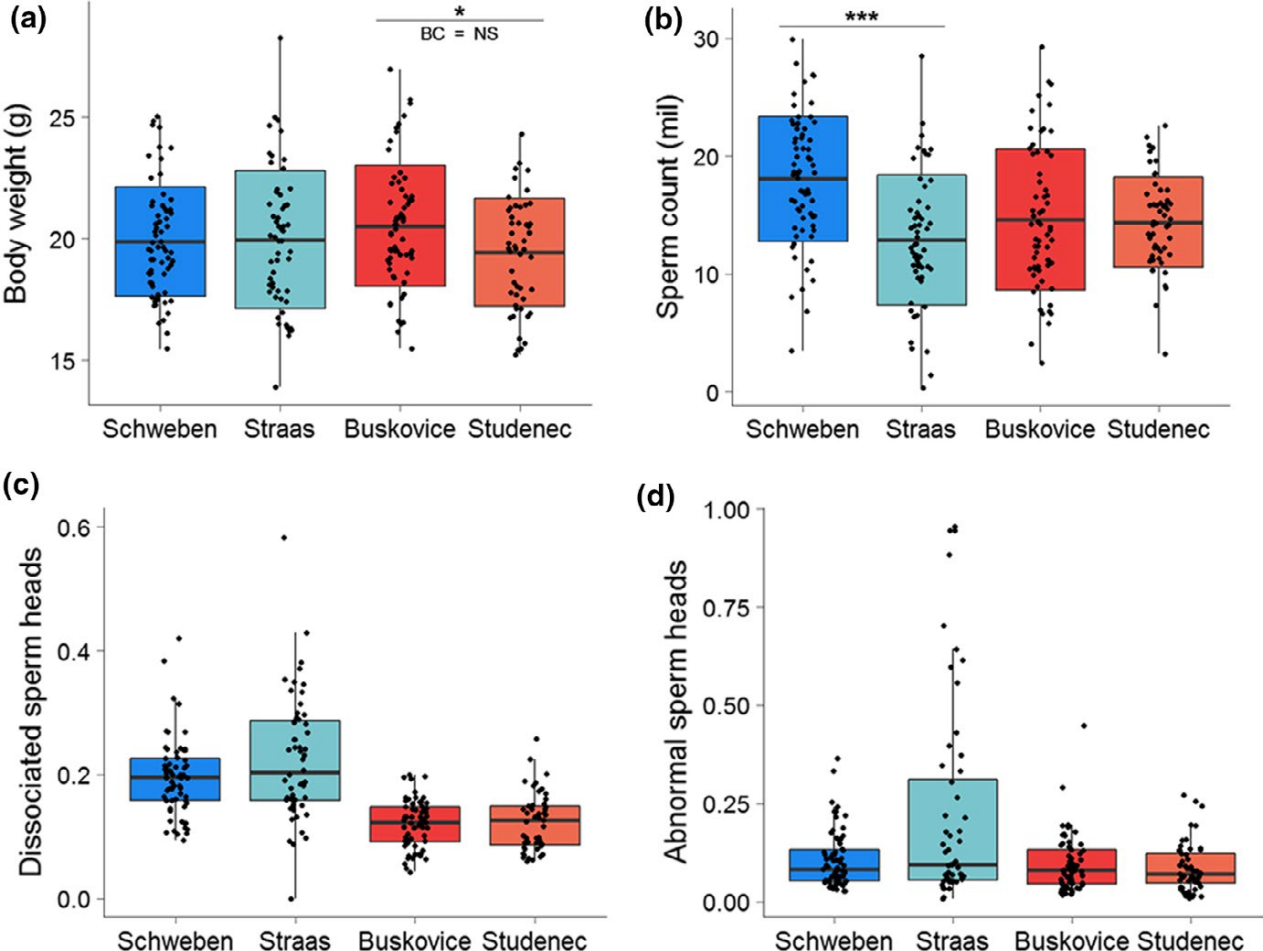
Boxplots summarizing variation in (a) body weight, (b) sperm count, (c) frequency of dissociated sperm heads, and (d) frequency of abnormally shaped sperm heads among males from two *domesticus* (left) and *musculus* (right) localities. In graphs a and b, the crossbars indicate the mean values, the box ranges display standard deviation, and whiskers depict the range between the maximum and minimum values. In graphs c and d, the crossbars refer to the medians, the boxes display the range between the 1st and 3rd quartile, and the whiskers span the 1.5 interquartile ranges. Black dots represent individual values. Graphs A and B show results of Welsh's *t* test, and graphs C and D show results of the Wilcoxon test. Lines and asterisks above the boxplots mark uncorrected significant differences between groups of males: “***” 0.001 “**” 0.01 “*” 0.5, BC = NS is displayed when such difference is not significant after the Bonferroni correction

Comparisons between different localities within the subspecies revealed a rather different pattern of phenotypic differentiation: When compared with the intersubspecific level, variation in sperm quality traits disappeared but significant differentiation was detected in the sperm count and body and testis weights. BW is the only phenotype displaying Y‐associated differentiation between the Y*^musculus^* localities. Y^Buskovice^ males were heavier than Y^Studenec^; however, the difference was not significant after the Bonferroni correction (Figure 3a). The between‐locality differences are most expressed in the sperm count where the Y^Straas^ males revealed, on average, lower values than both the Y^Studenec^ and Y^Buškovice^ males, whereas the Y^Schweben^ males produced by more than 5 × 10^6^ spermatozoa than the Y^Straas^ males and this value was above the SC averages observed in the Y^Studenec^ and Y^Buškovice^ males (Figure 4b). The same pattern was observed in TW (Data S5). It should be noted that SC and TW were significantly correlated (Spearman's correlation for Y*^domesticus^* and Y*^musculus^*, *r* = 0.76, *p* < 0.001 and *r* = 0.74, *p* < 0.001, respectively) this high correlation being present at all levels of the analysis hierarchy. Interestingly, the higher frequency of abnormal sperm heads in *domesticus* males than in *musculus* males appeared to be driven by the Y^Straas^ males, whereas the ASH median for the Y^Schweben^ males did not differ from both *musculus* localities (Figure 4d).

### 3.5 Interstrain effects (Y^WDS^ level)

**Figure 5:**
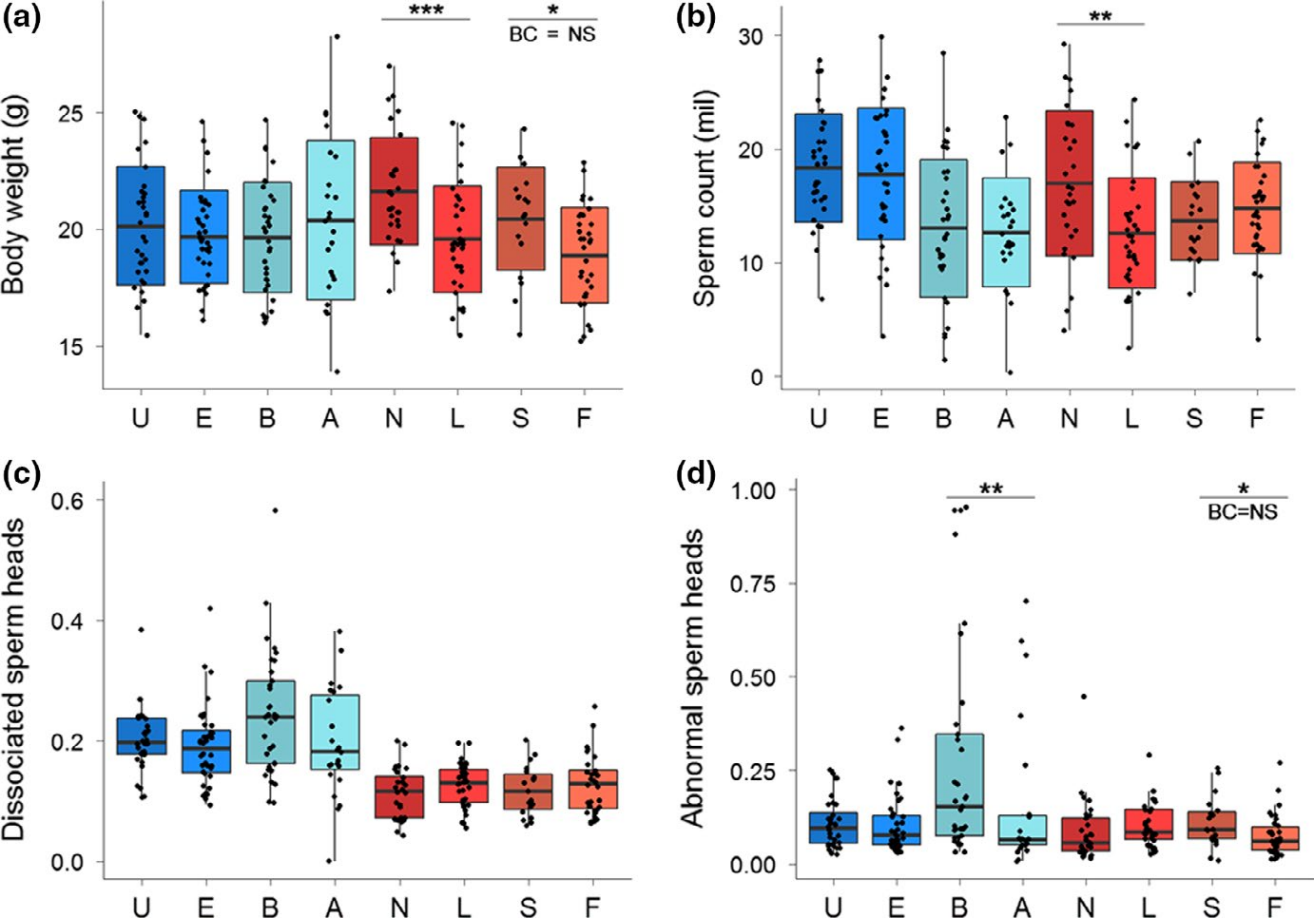
Boxplots for (a) body weight, (b) sperm count, (c) frequency of dissociated sperm heads, and (d) frequency of abnormally shaped sperm heads among the male groups with different Y chromosomes originating from the eight wild‐derived strains. One‐letter codes of strains are listed in Figure 1. In graphs a and b, the crossbars indicate the mean values, range of boxes display standard deviation, and whiskers give ranges between the maximum and minimum values. In graphs c and d, the crossbars refer to the medians, boxes display the 1st and 3rd quartile, and whiskers show the 1.5 interquartile ranges. Black dots represent individual values. Lines and asterisks above the boxplots mark significant differences between pairs. Graphs A and B show the results of Welsh's *t* test, and graphs c and d show the results of the Wilcoxon test. Lines and asterisks above boxplots mark uncorrected significant differences between groups of males: “***” 0.001 “**” 0.01 “*” 0.5, BC = NS is displayed when such difference is not significant after the Bonferroni correction

Splitting data according their Y^WDS^ origin revealed high variation in BW within the Y*^musculus^* males. This variation was especially pronounced between the two Y^Buškovice^ strains (Figure 5a), with the Y^BUSNA^ males (N) being by more than two grams heavier than the Y^BULS^ males (L), representing roughly 10% of their body weight. A similar trend was observed between the Y^STUS^ (S) and Y^STUF^ (F) males, although the difference (*p* = 0.02) was not significant when Bonferroni corrected (Figure 5a). The Y^BUSNA^ males showed a significantly higher mean sperm count than the Y^BULS^ males, the difference being as high as 4 × 10^6^ spermatozoa (Figure 5b). The Y^BUSNA^ and Y^BULS^ males differed also in SC and TW (*p* = 0.004 and *p* = 0.026, respectively); nevertheless, these differences were not significant after the Bonferroni correction (Figure 5b, Data S5). No intrastrain variation was detected in DSH (Figure 5c). These results seem to confirm systematic differentiation between the *domesticus* and *musculus* mice observed at the Y^ssp^ and Y^loc^ level analysis (cf. Figure 3c and 4c). Interestingly, the higher ASH variance in the Y^Straas^ males found at the Y^loc^ level was found to be driven by the significant difference between the two strains, where the Y^STRB^ males had a twice higher ASH frequency than Y^STRA^ males. However, this may not be a complete explanation since the variance of ASH was very high in the Y^STRB^ males themselves, much higher than in all other strains (Figure 5d). Although higher variation in ASH was detected in three pairwise WDS comparisons (*p* = 0.007–0.049), none of them remained significant after the Bonferroni correction.

### 3.6 Intrastrain effect (Y^RL^ level)

**Figure 6:**
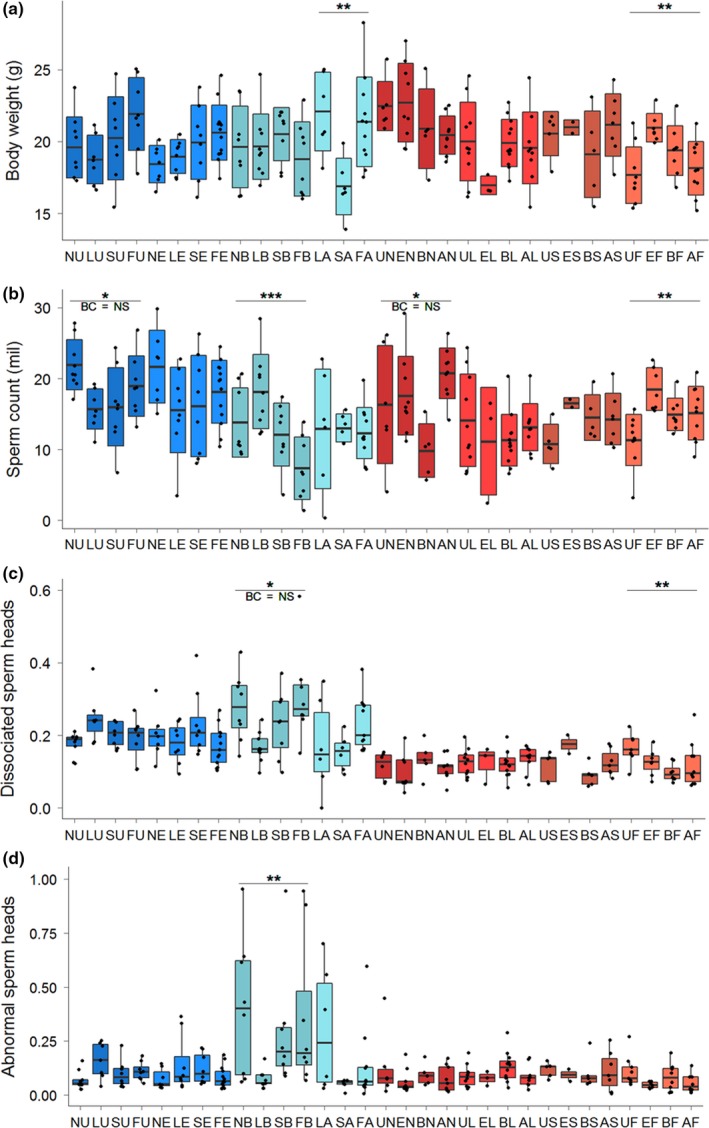
Boxplots distributions of (a) body weight, (b) sperm count, (c) frequency of dissociated sperm heads, and (d) frequency of abnormally shaped sperm heads among the 31 RLs with different Y chromosomes. Two‐letter codes of RLs are quoted in Figure 1. In graphs a and b, the crossbars indicate the mean values, box ranges display standard deviation, and whiskers give ranges between the maximum and minimum values. In graphs c and d, the crossbars refer to the medians, boxes display the 1st and 3rd quartile, and whiskers span the 1.5 ranges between the lower and upper quartiles. Black dots represent individual values. Lines and asterisks above boxplots mark significant differences within the groups. Graphs A and B show the results of Tukey's post hoc tests, and graphs c and d show the results of the Nemenyi post hoc tests. Lines and asterisks above the boxplots mark the uncorrected significant differences between groups of males: “***” 0.001 “**” 0.01 “*” 0.5, BC = NS is displayed when such difference is not significant after the Bonferroni correction

The analysis of intrastrain effects in 31 RLs differs from previous analyses in that the tests involve polymorphism in four RLs sharing the Y variant identical by descent. The most pronounced polymorphism in BW was detected in the group of Y^STRA^ males where the difference between two RLs (LA vs. SA, *p* = 0.006, not significant after the Bonferroni correction) appeared higher than 3 g, representing more than 20% of their BW (Figure 6a, Data S5). Significant differences in BW were found within the Y^STUF^ group where the observed variation in BW exceeded 15% of their BW (Figure 6a). While in majority of the Y^WDS^ groups BW in males from individual RLs spanned over and below the average of the whole dataset, the group of the Y^BUSNA^ males had BW consistently above the average.

Sperm count was the most variable phenotype. Four out of eight RLs clusters displayed significant differentiation among groups in each Y^WDS^ group (Figure 6b). In the Y^STRB^ and Y^STUF^ males, the intrastrain variation remained significant also after the Bonferroni correction. Interestingly, distribution of variation in SC was consistent across the localities of origin—while one group of Y^RL^ was homogeneous, the second one from the same locality was polymorphic. Maximum difference in SC reached almost 14.6 × 10^6^ between Y^FB^ and Y^NU^ males (Data S5). Despite significant correlation between TW and SC in the whole dataset (Spearman's correlation, *r* = 0.744, *p* < 0.001), variation in TW only partly mirrored that observed in SC, and only one of the Y^STUF^ males remained significant when Bonferroni adjusted (Data S5). DSH revealed significant (though not after the Bonferroni correction) differences in two of eight Y^RL^ clusters (Figure 6c).

Frequency of ASH displayed the lowest variation within the eight Y^RL^ clusters. The only significant differentiation was found in the Y^STRB^ group (*p* = 0.005, Figure 6d) where three Y^RL^ show ASH more than twice than is the frequency in the whole dataset (0.154). However, also this variation loses significance after the Bonferroni correction.

## 4 DISCUSSION

Studies on spread of genetic variants and inference on selection in hybrid zones are focused on interactions between two divergent (sub)species. However, as we showed in this paper reducing analyses on (sub)species‐specific comparisons and neglecting intra(sub)specific variation can lead to oversimplification of the reality. We have created a set of recombinant lines that sample natural genetic polymorphisms within two house mouse subspecies, and by their reciprocal crossings, we produced F1 hybrid males. In this way, we partitioned variation in a set of phenotypic traits directly related to fitness into subspecific, local, between‐strain, and within‐strain Y‐associated effects. Of the three expected alternatives mentioned in Introduction (i.e., consistency, asymmetry, and/or polymorphism in the Y‐associated effects in F1 hybrids), we found no support for the first one. On the contrary, we found compelling evidence for phenotypic polymorphism associated with Y chromosome variants. This variation was expressed diversely in different phenotypic traits and varied across the whole hierarchical scale. Before discussing the variable traits, however, we wish to make a note pointing to limits on data inference.

The way we mixed the genomes and generated the F1 hybrids can only model a very short period after establishment of the house mouse contact zone. Consequently, studies of differential phenotypic performance in these hybrids cannot be conclusive in inferring introgressive advantage of the associated Y variants on non‐native genetic backgrounds. On the other hand, the results can be considered as indicative for the potential of some Y‐linked phenotypes to perform better than others after the secondary contact. This differential would then be a prerequisite for their subsequent spread.

Another cautionary note relates to the strength of Y‐associated effects. We are aware that in all the traits under study, most of the variance remained unexplained (Table 1). The residual variance can derive from the Y‐X, Y‐autosomal, or cytonuclear interactions, from the complexity of the genetic basis of the scored phenotypes, and from the non‐heritable variance component in which the mice grew up. Nevertheless, the explained variation of Y‐associated effects did reveal some patterns opening a window to understanding the hybrid zone dynamics.

### 4.1 Asymmetry in the Y chromosome effects

Two of the traits showing differential phenotypic values were related to sperm quality. Both traits, the frequencies of dissociated and abnormal sperm heads (DSH and ASH, respectively), displayed increased values in Y*^domesticus^* compared to Y*^musculus^*. DSH was the only trait in which the mixed model explained more variance at the subspecific level (Y^ssp^) than at all other levels. The DSH medians were almost homogeneous on the lower scales. The increased variation in the recombinant lines bearing Y^STRB^ implies an interaction between the Y‐linked gene(s) and other autosomal/X‐linked loci. As far as we know, susceptibility toward dissociation of sperm heads from tails has been examined only marginally. Nevertheless, our results are in agreement with the strength and directionality of the effect obtained in the study of White, Stubbings, Dumont, and Payseur (2012): In crosses between the WSB (representing *domesticus*) and PWD (*musculus*) strain, the frequency of headless/tailless sperm was 0.38 in (PWD × WSB)F1, this value being significantly higher than 0.089 observed in the reciprocal F1 cross (White, Steffy, Wiltshire, & Payseur, 2011). Subsequent QTL mapping for headless/tailless sperm using the F2 recombinant progeny detected loci on chromosomes 15 and X; none of the QTL was mapped to the Y chromosome or mtDNA (White et al., 2012).

The higher incidence of ASH in sperm of the Y*^domesticus^* males compared to the Y*^musculus^* males was only significant without the Bonferroni correction. Most explained variance was detected among the RLs (11% of overall variance); then, it was almost equally partitioned to the subspecific and strain levels (9% of overall variance). As almost 80% of variance remains unexplained, the Y‐associated effects cannot be explanatory variables themselves and they rather seem to interact with other quantitative trait loci. Hierarchical top‐down splitting of the variation revealed that the intersubspecific differentiation is driven through markedly increased variance of ASH in the Y^Straas^ males (Figure 4d). Increased frequencies of ASH between the Y^STRA^ and Y^STRB^ males document the presence of polymorphism within a single locality. Interestingly, the STRA and STRB Y chromosomes share the same SNP alleles so the results can be interpreted as ASH being only partly affected by the scored Y‐linked loci. Contrary to DSH, the Y‐associated effects on ASH are a common phenomenon in mice. These have been reported in a variety of diverse crosses such as the KE (unknown origin) and CBA (classical laboratory strain of the *domesticus* origin, carrying the Y*^musculus^*) strains (Krzanowska, 1969), B10.BR/SgSn (classical laboratory strain of the *domesticus* origin, carrying the Y*^musculus^*) and its congenic mutant strain B10.BR‐Ydel, with a partial deletion in the Y chromosome (Styrna, Imai, & Moriwaki, 1991), the outbred albino MF1 strain (Ellis et al., 2005), and more recently also in the wild‐derived strains PWK, PWD (both of the *musculus* origin), LEWES, and WSB (both of the *domesticus* origin) (Campbell, Good, Dean, Tucker, & Nachman, 2012; Campbell & Nachman, 2014; Good, Dean, & Nachman, 2008; White et al., 2011). Strong evidence of subspecific modulation of ASH frequency was reported in a cross‐utilizing three strains (Larson et al., 2018) where females of the WSB strain were mated with males of two *musculus* strains, PWK and CZECHII, and the two types of produced F1 hybrids significantly differing in the proportion of ASH. In a test cross between WSB females and (PWK × CZECHII)F1 and (CZECHII × PWK)F1 males, the QTLs contributing to low sperm counts and abnormal sperm morphology were detected on various autosomes. Unfortunately, no data on X‐, Y‐, and mtDNA‐linked QTLs were presented (Larson et al., 2018). Deletions in the long arm of the Y chromosome and RNA interference indicate that the *Sly* gene is a causal link to sperm head deformities (Case et al., 2015; Cocquet et al., 2009, 2012). Along with the detected Y‐linked genetic correlates, several studies associated ASH frequency with mechanisms affecting male fitness. For example, in vivo examinations of the ability of abnormal sperm to reach fertilization suggest the uterus junction as a barrier preventing deformed sperm reaching the eggs (Krzanowska, 1974; Nestor & Handel, 1984).

In summary, the frequency of dissociated sperm heads was the only trait differ significantly the Y*^domesticus^* relative to the Y*^musculus^* males. The direction of asymmetry is in agreement with the observed introgression of the Y*^musculus^* chromosomes onto *domesticus* background as demonstrated in many replicates of Central European hybrid zone (Ďureje et al., 2012), in western Norway (Jones et al., 2010) or in the majority of classical laboratory strains that carry the *molossinus*/*musculus* Y type (Bishop, Boursot, Baron, Bonhomme, & Hatat, 1985; Yang et al., 2011). Differentiation of abnormal sperm heads at subspecific level was weakly supported and attributed to one strain. We conclude that while frequencies of dissociated sperm heads have potential to affect the dynamics of the Y behavior in the house mouse hybrid zone, the frequencies of deformed sperm heads will be subject of interactions with other loci and can affect the spread of the Y only locally.

### 4.2 Polymorphism in Y chromosome effects

Although no subspecific effects were detected in sperm count (SC) and body weight (BW), these traits displayed substantial variation at the intrasubspecific (both between and within localities) and intrastrain levels. Here, we will point to three phenomena connected with this variation. First, as anticipated, the absence of intersubspecific differentiation can result from pooling data of opposing effects from distinct localities. For example, averages of BW were found to be almost identical between the Y*^domesticus^* and Y*^musculus^* individuals. However, at lower hierarchical levels this trait started to display different patterns of variation. Whereas BW was significantly differentiated both between and within *musculus* localities, it was almost homogeneous between and within Y*^domesticus^* localities and increased variation only appeared on the lowest, Y^RL^, scale (especially within the group of the Y^STRA ^males).

Second, for the spread of a Y variant across a population it is necessary this variant to be associated with a phenotype that will perform better than the less fit variant being ultimately replaced. Regarding the BW data, such fitness differences were observed at both the WDS and RL levels where the Y^BUSNA^ males represented the heaviest group of hybrids. As BW is a determinant of male competitive advantage, BW may facilitate the spread of the Y^BUSNA^ chromosomes. The direct effect of the Y chromosome on male body weight seems to be corroborated by the high proportion of explained variation (23% of overall variance). Nevertheless, high differentiation within the four recombinant lines sharing the same Y (strongest among the Y^STRA^ and Y^STUF^ males, respectively; Figure 6a) indicates that also interactions with other genomic regions probably shape the observed phenotypic variation. Indeed, a picture emerging from other studies suggests that BW is a polygenic trait with QTLs scattered across the whole genome (Chan et al., 2012; Corva & Medrano, 2001) and may also be partly under the control of the Y chromosome. Using a panel of 17 Y chromosome consomic strains sharing the same genetic background, a continuous distribution in body weight in adult mice was identified (Suto, 2013). As body weight was independent of the autosomal and X chromosome genetic background, the results were interpreted that Y the chromosome contains genes contributing to body size in mice.

Third, the spread of advantageous variants for any trait can be context‐dependent. For example, pronounced variation in SC was observed within the Y*^domesticus^* males. Males possessing the Y^BUSNA^ chromosome may outcompete males with the Y^STRA^ or Y^STRB^ chromosomes due to higher sperm count whereas this invasion could be prevented in regions occupied by males carrying the Y^SCHUNT^ or Y^SCHEFE^ chromosomes (Figure 5b). This suggests that while a Y variant can invade a territory of a less fit Y variant, its spread will be hampered at regions occupied by a fitter variant (or, in the case of encounter of two equally fit Ys, we may expect their symmetric diffusion). However, this simplistic scenario will be modulated by Y‐X and Y‐autosomal interactions as well as by recombination rates between the interacting loci. There is an empiric observation of such context‐depending Y spread.

It has been shown that in the Czech‐Bavarian portion of the zone, the Y*^musculus^*, which introgresses into the *domesticus* genome, rescues sperm numbers in comparison with *domesticus* males with their native Y chromosomes (Albrechtova et al., 2012). The invasive front of Y*^musculus^* is sharply delimited by an abrupt cline from Y*^domesticus^* (Macholán et al., 2008). This suggests the advantageous variant reached its spread limits finding a Y*^domesticus^* variant resistant to replacement. The spread is also modulated by genetic conflict detected in the same hybrid zone. The Y invasion is associated with restoration of sex ratio of female‐biased distortion in noninvaded *domesticus* and *domesticus* populations close to the hybrid zone, pointing to an ongoing genetic conflict between Y and X chromosomes (Macholán et al., 2008, 2011). Both sex chromosomes in mice carry ampliconic gene families, including *Slx/Slx1* and *Sly*, whose copy numbers vary between and even within the subspecies (Cocquet et al., 2009; Ellis, Bacon, & Affara, 2011; Scavetta & Tautz, 2010). Those genes have an antagonistic effect during sperm differentiation, and they are involved in a postmeiotic intragenomic conflict that causes segregation distortion, abnormal spermatogenesis, and hybrid sterility. This situation is expected when balance between *Slx/Slxl1* and *Sly* copy numbers, and therefore expression, is disrupted (Cocquet et al., 2009, 2010, Cocquet et al. 2012).

As noted above, in F1 hybrids it is hard to distinguish Y‐associated phenotypic effects from effects of mtDNA. Nevertheless, we can provide arguments why the Y is more likely to contribute to the phenotypic variation observed in this study than the mitochondrial genome. At the intersubspecific level (Y^ssp^), significances of mtDNA effects on DSH would be the same as for the Y; however, the direction of the effect would be opposite; that is, the frequencies of DSH would be higher in the mtDNA*^musculus^* males (having Y*^domesticus^*) than in the mtDNA*^domesticus^*/Y*^musculus^* males. This would predict an advantage for the mtDNA*^domesticus^* variant yet it is unclear how this advantage would pass from fathers to sons as mtDNA is transmitted maternally. Mitochondrial DNA is known to introgress across the house mouse hybrid zone in either direction (Božíková et al., 2005). This would imply the existence of differentiation in mtDNA‐associated phenotypic variation; however, no Bonferroni corrected significant mtDNA effects at the intrasubspecific level, and only one interstrain effect in the mtDNA^Straas^ males, were detected. Finally, to the best of our knowledge, most of the mtDNA effects reported in the literature are associated with sperm motility and not with the traits analyzed in this study (for a review, see St. John, Jokhi, & Barratt, 2005).

Asymmetry and polymorphism in Y chromosome phenotypic effects was also revealed in studies of mouse hybrid sterility based on crosses of a vast array of *musculus* and *domesticus* strains (Britton‐Davidian, Fel‐Clair, Lopez, Alibert, & Boursot, 2005; Forejt & Iványi, 1974; Good et al., 2008; Larson et al., 2018; Vyskočilová, Pražanová, & Piálek, 2009; Vyskočilová et al., 2005; White et al., 2011). In the present study, we did not detect any fully sterile individuals (no sperms in epididymis) in the group of 240 RL males (the lowest sperm count, 0.35 × 10^6^, was scored in a Y^LA^ male, Figure 6b). We also did not detect any considerably higher proportion of oligospermatic (abnormally low sperm count) males: There were only nine (0.04%) individuals with SC < 5 × 10^6^ in the whole sample. Of these males, three were Y^FB^ males, nevertheless individuals carrying the Y^STRB^ chromosomes significantly segregated for SC (Figure 5b): For example, the mean difference between the Y^FB^ and Y^LB^ males was 10.6 × 10^6^ (see Data S5). We can thus conclude that hybrid male sterility was not substantially represented in these F1 hybrids. Nevertheless, lack of difference in sperm count at subspecific level documented here suggests that the spread of the Y*^musculus^* in the Czech replicate (Albrechtova et al., 2012) may not be universally present along the whole length of the hybrid zone.

Genetic polymorphism in Y chromosomes can be a clue for understanding the heterogeneity of opinions regarding introgressive behavior of the Y chromosome in the hybrid zone noted in Introduction. However, empirical and theoretical studies of the Y chromosome dynamics have usually been focused on binomial markers fixed for alternative variants in respective subspecies (here marked as the Y*^domesticus^* and Y*^musculus^* variants). For example, most studies assessing the behavior of the Y in the mouse contact zone used loci within the non‐recombining region such as the *Zfy2* gene (Macholán et al., 2008; Munclinger et al., 2002) and/or DNA restriction patterns (Tucker et al., 1992; Vanlerberghe et al., 1986). Similarly, simulation studies modeling genetic incompatibilities between sex chromosomes and autosomes have been based on diagnostic alleles (e.g., Sciuchetti et al., 2018). Most of genetic polymorphism detected here can be also classified as subspecies‐specific variants. Nevertheless, we found a signal for intrasubspecific variation in almost 8% of SNPs. Within each subspecies, this polymorphism was extended to the local level and each locality had its own specific haplotype.

Ultimate knowledge on Y genetic variation can be obtained from long‐range sequencing. The Y chromosome is notoriously known for problems with sequencing due to low complexity regions and high copy number variation especially in the long Yq arm (Soh et al., 2014). Sequenced Y chromosomes also reveal the presence of high variation in SNPs, copy number variation, and small indels comparable with other genomic segments between and within mouse subspecies (Harr et al., 2016; Keane et al., 2011; Morgan & de Villena, 2017; Scavetta & Tautz, 2010). This variability is a prerequisite for the evolution of different functional behavior among different house mouse Y haplotypes; however, associating Y genetic variation with phenotypic variation is under explored in mice. Such studies appeared challenging in Norway rats where the role of genetic variants and gene duplications was explored in multiple Y consomic strains. Sequencing of the male‐specific region of chromosome Y (MSY) revealed that (a) genetic variation altering a broad range of inbred rat phenotypes and (b) per chromosome size, MSY contributed to higher strain‐specific male phenotypic variation relative to all other chromosomes (Prokop et al., 2016).

### 4.3 Perspective

In this study, we admixed 4 *domesticus* and 4 *musculus* genomes separately within each subspecies. This design allowed, on average, two recombination events per chromosome within each subspecies. After generating intersubspecific F1 hybrids, we found that incorporating eight strains with four SNP‐defined Y haplotypes into the experimental crossing had a dramatic influence on phenotypic variation between and within mouse subspecies. Despite this effect, the introduced genetic variation and number of recombination events are only a tiny fraction of that which is expected to exist in hybrid zones. This finding adds another dimension of complexity to studies of Y introgression not only across the house mouse hybrid zone but potentially also in other secondary contact zones.

Analyses of various replicates of this zone have frequently built on a reduced one‐dimensional model based on distances from the zone center and introgression being measured as a cline shift of a locus in either direction (review in Baird & Macholán, 2012). Such reduction of two‐dimensional sampling (defined by geographic coordinates) prevents plausible description of the extent of Y introgression whose cline orientation can be different from the consensus center of hybrid zone (Macholán et al., 2008). To specify further the dynamics of the Y chromosome behavior in the zone, sampling design was enlarged to an area from the Bavarian Alps to the Baltic Sea coast (Ďureje et al., 2012). Currently, we are analyzing molecular genetic data to localize Y introgression along this contact. Results of this study suggest that Y introgression will be predominantly unidirectional and polymorphic between geographic replicates. Again, contrary to one‐dimensional analytical models, the spread of an advantageous variant will be facilitated in two‐dimensional space offering more sites to cross the barrier (Piálek & Barton, 1997). Given the length of the house mouse hybrid zone in Central Europe, there is an ample space for advantageous variants to cross. The molecular data will clarify whether Y introgression is from a single resource or if multiple Y variants can invade the *domesticus* background.

## AUTHORS CONTRIBUTION

IM conducted experimental crossing, animal maintenance, data collection, analysis, and visualization. LD participated in data collection. JK participated in data analysis. MM contributed to the result interpretation. JP designed the study. IM and JP wrote the manuscript with contribution of all other authors.

## DATA AVAILABILITY STATEMENT

All data and the R script used in this manuscript are available as electronic supplementary material.
